# A review of national health surveys in India

**DOI:** 10.2471/BLT.15.158493

**Published:** 2016-02-12

**Authors:** Rakhi Dandona, Anamika Pandey, Lalit Dandona

**Affiliations:** a Public Health Foundation of India, Plot 47, Sector 44, Gurgaon – 122 002, National Capital Region New Delhi, India.

## Abstract

Several rounds of national health surveys have generated a vast amount of data in India since 1992. We describe and compare the key health information gathered, assess the availability of health data in the public domain, and review publications resulting from the National Family Health Survey (NFHS), the District Level Household Survey (DLHS) and the Annual Health Survey (AHS). We highlight issues that need attention to improve the usefulness of the surveys in monitoring changing trends in India’s disease burden: (i) inadequate coverage of noncommunicable diseases, injuries and some major communicable diseases; (ii) modest comparability between surveys on the key themes of child and maternal mortality and immunization to understand trends over time; (iii) short time intervals between the most recent survey rounds; and (iv) delays in making individual-level data available for analysis in the public domain. We identified 337 publications using NFHS data, in contrast only 48 and three publications were using data from the DLHS and AHS respectively. As national surveys are resource-intensive, it would be prudent to maximize their benefits. We suggest that India plan for a single major national health survey at five-year intervals in consultation with key stakeholders. This could cover additional major causes of the disease burden and their risk factors, as well as causes of death and adult mortality rate estimation. If done in a standardized manner, such a survey would provide useable and timely data to inform health interventions and facilitate assessment of their impact on population health.

## Résumé

**Les différentes séries d'enquêtes nationales sur la santé menées depuis 1992 en Inde ont généré d'importants volumes de données. Dans cet article, nous décrivons et comparons les informations sanitaires clés recueillies, évaluons la disponibilité des données sanitaires dans le domaine public et examinons les publications issues de l'enquête nationale sur la santé des familles (NFHS, *National Family Health Survey*), de l'enquête réalisée auprès des ménages à l'échelle du district (DLHS, *District Level Household Survey*) et de l'enquête annuelle sur la santé (AHS, *Annual Health Survey*). Nous mettons en avant les questions à traiter pour renforcer l'utilité des enquêtes et pouvoir suivre l'évolution des tendances concernant la charge de morbidité en Inde: (i) intérêt insuffisant porté aux maladies non transmissibles, aux blessures et à certaines des principales maladies transmissibles; (ii) faible comparabilité entre les enquêtes sur les thèmes essentiels de la mortalité maternelle et infantile et de la vaccination permettant de comprendre les tendances dans le temps; (iii) intervalles courts entre les dernières séries d'enquêtes; et (iv) retards dans la mise à disposition des données individuelles pour analyse dans le domaine public. Nous avons identifié 337 publications qui utilisaient les données de l'enquête NFHS, contre seulement 48 et 3 qui utilisaient respectivement les données de l'enquête DLHS et de l'enquête AHS. Étant donné que les enquêtes nationales nécessitent d'importantes ressources, il serait prudent d'en optimiser les bénéfices. Nous suggérons que l'Inde prévoie une seule grande enquête nationale sur la santé tous les cinq ans, en consultation avec les principaux acteurs concernés. Celle-ci pourrait s'intéresser aux autres causes importantes de la charge de morbidité et à leurs facteurs de risque, ainsi qu'aux causes de décès, et réaliser une estimation du taux de mortalité des adultes. Si elle est effectuée de façon standardisée, cette enquête pourrait fournir des données utilisables et récentes permettant d'orienter les interventions en matière de santé et faciliter l'évaluation de leur impact sur la santé de la population:**

## Resumen

Varias rondas de encuestas nacionales de salud han generado una gran cantidad de datos en India desde 1992. Se describe y compara la información sanitaria fundamental recopilada, se evalúa la disponibilidad de datos sanitarios de dominio público y se revisan publicaciones derivadas de la Encuesta Nacional de Salud Familiar (NFHS, por sus siglas en inglés), la encuesta de las Instalaciones a Nivel de Distrito (DLHS, por sus siglas en inglés) y la Encuesta Anual de Salud (AHS, por sus siglas en inglés). Se destacan los asuntos que requieren atención para mejorar la utilidad de las encuestas en cuanto al control del cambio de tendencias de las enfermedades en India: (i) cobertura inadecuada de enfermedades no contagiosas, daños y algunas enfermedades contagiosas importantes; (ii) modesta comparación entre encuestas sobre temas fundamentales de mortalidad infantil y materna e inmunización para comprender las tendencias a lo largo del tiempo; (iii) intervalos cortos entre las rondas de encuestas más recientes; y (iv) retrasos para obtener datos individuales para analizar en el dominio público. Se identificaron 337 publicaciones utilizando datos de la NFHS, en contraste con las 48 y 3 publicaciones que utilizaban datos de la DLHS y la AHS, respectivamente. Puesto que las encuestas nacionales exigen gran cantidad de recursos, sería prudente multiplicar sus beneficios. Nuestra sugerencia es que India planifique una única encuesta nacional sobre salud en intervalos de cinco años en consulta con las principales partes interesadas. Esto podría abarcar más causas principales de las enfermedades y sus factores de riesgo, así como las causas de muerte y el cálculo de las tasas de mortalidad adulta. Si se realiza de forma estandarizada, dicha encuesta puede ofrecer información útil y oportuna para informar sobre intervenciones sanitarias y facilitar la evaluación de su impacto en la salud pública.

## ملخص

تمخضت جولات عديدة من الدراسات الاستقصائية الوطنية عن التوصل إلى كم هائل من البيانات في الهند منذ عام 1992. وإننا نعمل على شرح ومقارنة المعلومات الصحية الأساسية التي تم جمعها، وتقييم مدى إتاحة البيانات الصحية للملكية العامة، ومراجعة المطبوعات التي تمثل نتاجًا للدراسة الاستقصائية الوطنية المعنية بصحة الأسرة (NFHS)، والدراسة الاستقصائية للأسر المعيشية على مستوى المقاطعات (DLHS)، والدراسة الاستقصائية السنوية المعنية بالصحة (AHS). كما نلقي الضوء على القضايا التي تستوجب الالتفات إليها لرفع مستوى جدوى الدراسات الاستقصائية فيما يتعلق بمراقبة اتجاهات التغير في عبء المرض في الهند: القصور في تغطية الأمراض غير المعدية، والإصابات، وبعض الأمراض الرئيسية المعدية؛ و(2) إمكانية إجراء مقارنة بسيطة بين الدراسات الاستقصائية المتعلقة بالموضوعات الأساسية بشأن الوفيات عند الأطفال والأمهات وتحصينهم ضد العدوى لفهم اتجاهات ذلك على مدى فترات من الزمن؛ و(3) قصر الفترات الزمنية الفاصلة بين مرات إجراء أحدث الدراسات الاستقصائية؛ و(4) فترات التأخر في إتاحة البيانات على المستوى الفردي للملكية العامة بغرض تحليلها. وحددنا 337 مطبوعة تستخدم بيانات الدراسة الاستقصائية الوطنية المعنية بصحة الأسرة، وعلى النقيض استخدمت 48 مطبوعة وثلاث مطبوعات فقط البيانات الواردة في الدراسة الاستقصائية للأسر المعيشية على مستوى المقاطعات والدراسة الاستقصائية السنوية المعنية بالصحة على التوالي. ونظرًا لغنى الدراسات الاستقصائية الوطنية بالمصادر تقتضي الحكمة تحقق أقصى استفادة منها. ونقترح وضع الهند لخطة بغرض إجراء دراسة استقصائية وطنية رئيسية واحدة عن الصحة بفترة زمنية فاصلة تبلغ خمس سنوات، وذلك بالتشاور مع الجهات المعنية الرئيسية. وقد يشمل هذا الأمر المزيد من الأسباب الرئيسية لعبء المرض وعوامل المخاطرة المرتبطة بها، وكذلك الأسباب المؤدية للوفاة، وتقدير معدل الوفيات بين الراشدين. وإذا تم ذلك بأسلوب يراعي المعايير، فستقدم تلك الدراسة بيانات في الوقت المناسب تصلح للاستخدام من أجل تقديم المعلومات اللازمة لإجراء التدخلات الصحية وتيسير تقييم آثارها على صحة السكان.

## 摘要

自 1992 年起，印度展开了多次全国健康调查，从而生成大量数据。我们描述并比较收集到的关键健康信息，评估健康数据在公共领域的可用性，并且评审依据全国家庭健康调查 (NFHS)、地区家庭调查 (DLHS) 和年度健康调查 (AHS) 数据而产生的出版物。我们强调需要注意的问题，以提升监控印度疾病负担改变趋势方面调查的有用性：(i) 非传染性疾病、受伤和一些主要传染性疾病的覆盖面不足；(ii) 以孩子和产妇死亡率为关键主题的调查和以免疫力为关键主题的调查之间的适度可比性，可以了解随时间发展的趋势；(iii) 最近几次调查的间隔时间较短；以及 (iv) 让个体层面的数据用于公共领域内分析有所拖延。我们利用 NFHS 数据确定了 337 种出版物，相比之下，分别利用 DLHS 和 AHS 数据确定的出版物只有 48 种和 3 种。由于全国调查占用大量资源，因此需谨慎地在最大程度上发挥其效益。我们建议印度与关键的利害关系人协商，为每隔五年展开一次主要全国健康调查制定计划。这可能涵盖疾病负担的额外主要致因和风险因素，以及死因和成人死亡率评估。如果以标准化的方式进行，此类调查将提供及时有用的数据，告知健康干预并促进对人口健康影响的评估。

## Резюме

В результате серии национальных исследований состояния здоровья, проводимых в Индии с 1992 года, было получено огромное количество информации. В статье описываются и сравниваются основные полученные сведения о состоянии здоровья, оценивается доступность подобных сведений, находящихся в общественной собственности, и анализируются публикации, составленные на основе данных Национального исследования состояния здоровья семей (NFHS), Окружного исследования домашних хозяйств (DLHS) и Ежегодного исследования состояния здоровья (AHS). На первый план выдвигаются проблемы, которые требуют внимания и решение которых позволит повысить практическую ценность исследований для отслеживания меняющихся тенденций в бремени заболеваний в Индии. В их число входят: (i) недостаточный охват неинфекционных заболеваний, травм и некоторых основных инфекционных заболеваний; (ii) недостаточная сопоставимость исследований на такие основные темы, как детская и материнская смертность и иммунизация, для понимания тенденций во временной динамике; (iii) небольшие интервалы между последними раундами исследований; (iv) запоздалый переход данных индивидуального уровня в общественное достояние для их анализа. Были определены 337 публикаций, основывающихся на данных NFHS. Для сравнения: лишь 48 и 3 публикации были составлены на основании данных DLHS и AHS соответственно. Поскольку для проведения национальных исследований требуются значительные ресурсы, целесообразно извлекать из них максимальную пользу. Авторы статьи предлагают Индии разработать единое крупное национальное исследование состояния здоровья населения, которое проводилось бы с интервалом в пять лет при консультативной поддержке со стороны основных заинтересованных лиц. Такое исследование могло бы охватить ряд основных причин бремени заболевания и их факторы риска, а также причины смерти и оценку уровня смертности взрослого населения. Такое исследование, проводимое в соответствии с единым стандартом, позволило бы получать ценную и актуальную информацию, необходимую для мероприятий здравоохранения и упрощающую оценку их влияния на здоровье населения.

## Introduction

Health information gathering is an important part of any health system, but is often weak in low-income countries, plagued by poor quality data that are inadequate for informing health policy.^1^^–^^4^

Population-based surveys are an invaluable source of health information.^5^ A key aim of these surveys is to provide high-quality data for policy development and programme planning, monitoring and evaluation. Population-based surveys have been used extensively to gather information on fertility, mortality, family planning, maternal and child health, and some other aspects of health, nutrition and health care in India.^6^

We have previously reported that the health information system in India has not kept up with the epidemiological transition.^6^ In this paper, we assess national health surveys conducted in India since 1992 that were designed to provide information on health indicators at subnational levels. We describe and compare the health information covered by these surveys over time, the availability of resulting data in the public domain and the use of these survey data in publications. Based on our findings, we highlight the issues that need consideration to improve the usefulness of these surveys. We believe they should be able to provide more effective, useable and timely data on the health status of the population, given the evolving disease burden in India.

## Reviewing surveys

We selected large-scale, national, population-based household surveys that provided data on health indicators at the subnational levels in India from 1992 to 2015. These were the National Family Health Survey (NFHS), the District Level Household Survey (DLHS) and the Annual Health Survey (AHS), (Box 1). The surveys are summarized in Table 1.

Box 1 **Description of major surveys done in India between 1992 and 2016**

The National Family Health Survey (NFHS) is the equivalent of demographic and health surveys done in many countries around the world.^7^ The NFHS is overseen by the Ministry of Health and is coordinated by the International Institute for Population Sciences (IIPS) in Mumbai, as the nodal agency, with support from ORC Macro and other agencies.^8^ The primary aim of the NFHS has been to provide information on maternal and child health and reproductive health. Three rounds of the NFHS were conducted in 1992–1993, 1998–1999 and 2005–2006, and the fourth round is currently underway.^9^^–^^12^ The first three rounds of the NFHS were designed to provide state level data, but the fourth round, with a much larger sample size, will generate estimates of most indicators for all 640 districts in the country.^12^

The District Level Household Survey (DLHS) was launched in response to the need for district-level data on the Reproductive and Child Health Programme.^13^ The DLHS is carried out by the International Institute for Population Sciences with oversight by the Ministry of Health. Four rounds of DLHS have been undertaken: 1998–1999, 2002–2004, 2007–2008 and 2012–2014.^14^^–^^17^ The fourth round was done in coordination with the Annual Health Survey (AHS), with the former not conducted in nine states covered by the latter. The AHS has been conducted in the less developed states of India (Assam, Bihar, Chhattisgarh, Jharkhand, Madhya Pradesh, Odisha, Rajasthan, Uttar Pradesh and Uttarakhand).^18^ The sample sizes at the district level in the AHS are much larger than those in the DLHS and aim to generate more robust estimates at the district level, especially of infant mortality. The AHS is implemented by the Office of the Registrar General of India with funding from the Ministry of Health. The baseline round of the AHS was undertaken in 2010–2011, while two subsequent rounds in 2011–2012 and 2012–2013 collected data on the same households as in the baseline.^19^^–^^21^ In contrast, the DLHS and the NFHS have new cross-sectional samples for each round.

**Table 1:** Survey and sample size for major health surveys in India, 1992 to 2016

Survey	Survey years	No. of households in the sample
**NFHS**		
NFHS-1	1992–1993	88 562
NFHS-2	1998–1999	91 196
NFHS-3	2005–2006	109 041
NFHS-4^a^	2015–2016	568 200
**DLHS**		
DLHS-1	1998–1999	529 817
DLHS-2	2002–2004	620 107
DLHS-3	2007–2008	720 320
DLHS-4^b^	2012–2014	350 000
**AHS^b^**		
AHS baseline	2010–2011	4 140 000
AHS 1st update	2011–2012	4 280 000
AHS 2nd update	2012–2013	4 320 000

AHS: Annual Health Survey; DLHS: District Level Household Survey; NFHS: National Family Health Survey.

^a^ Data collection is ongoing as of February 2016.

^b^ DLHS-4 covered 336 districts in 26 states and union territories of India. The AHS covered 284 districts in the other nine states of India.

### Themes

We reviewed the survey questionnaires to assess: survey period and sample sizes; types of respondent; key themes; timeframe for availability of data in the public domain; and analytical publications resulting from the data. A more detailed review of the number of children, reference period and age groups was undertaken to gauge the utility of the data for assessment of trends in child mortality, maternal mortality and immunization.

To determine how well the household, male and female survey questionnaires corresponded to disease burden in the country, we assessed the proportion of questions covering major themes: maternal and child health; reproductive health other than infections; reproductive tract infections; other adult infections; noncommunicable diseases; and injuries. Data on anthropometric and biological markers were analysed in addition to the questionnaires.

We recorded the time between the completion of data collection for each survey round and the availability of individual-level data in the public domain. We conducted a PubMed database search to identify peer-reviewed research papers from January 1993 to March 2015 that had used data from either or all of the first three rounds of the NFHS and DLHS. For the AHS, this search was done for research papers published between January 2011 and March 2015. The fourth rounds of the NFHS and DLHS were not included in this search as the data collection for the former is not yet complete and the data for the latter have not yet been released in the public domain. The search terms used in PubMed to identify relevant publications were “National Family Health Survey” or “NFHS and India”, “District Level Household Survey” or “DLHS and India” and “Annual Health Survey” or “AHS and India”. We screened the titles and abstracts of identified articles and reviewed the full texts of those that analysed data from the surveys. Review papers and the papers that merely made reference to survey data in background or discussion sections were excluded.

## Survey characteristics

### Survey period

The first survey conducted was NFHS-1 in 1992–1993. The following three rounds of NHFS were done in 6–9 year intervals, which were longer than the DLHS interval of 4–5 years. The period of the first DLHS survey overlapped with NFHS-2 and the following survey rounds done with close proximity. The AHS, which is complementary to DLHS-4, was initially done in 2010–11, with two further rounds between 2011 and 2013 (Table 1).

### Types of respondents

There were some changes in the types of respondents across these surveys over time (Table 2). Ever-married women were surveyed in all rounds of the NFHS and AHS. DLHS-1 and DLHS-2 surveyed only currently married women but DLHS-3 and DLHS-4 surveyed ever-married women. NFHS-3, NFHS-4 and DLHS-3 also included never-married women.

**Table 2:** Types of respondents and key themes identified in major health surveys, India, 1992 to 2016

Survey	Household		Women		Men	
	Respondent	Key survey themes	Respondent	Key survey themes	Respondent	Key survey themes
**NFHS**						
NFHS-1	Household head	– Sociodemographic characteristics – Household amenities – Morbidity – Mortality (all ages)	Ever-married women 13–49 years of age	– Birth history – Maternal and child health – Child mortality – Family planning and fertility preferences – Woman and husband’s background characteristics – Women’s employment status	Not included in survey	N/A
NFHS-2	Household head	– Sociodemographic characteristics – Household amenities – Morbidity and risk factors – Health care use – Mortality (all ages)	Ever-married women 15–49 years of age	– Birth history – Maternal and child health – Child mortality – Family planning and fertility preferences – Woman and husband’s background characteristics – Women’s employment status – Women’s autonomy and domestic violence – Quality of health services – STIs and HIV/AIDS	Not included in survey	N/A
NFHS-3	Household head	– Sociodemographic characteristics – Household amenities – Child labour – Morbidity – Health care use	Ever-married women 15–49 years of age and never-married women 15–49 years of age	– Birth history – Maternal and child health – Child mortality – Family planning and fertility preferences – Woman and husband’s background characteristics – Women’s employment status – Women’s autonomy and domestic violence – Quality of health services – STIs and HIV/AIDS – NCDs and behavioural risk factors – Use of ICDS – Marital and sexual relationships and living arrangements	Men 15–54 years of age	– Reproductive history – Marital and sexual relationships, and living arrangements – Family planning and fertility preferences – Male involvement in health care – Quality of health services – NCDs and behavioural risk factors – Attitude towards women’s autonomy and domestic violence – STIs and HIV/AIDS
NFHS-4	Household head	– Sociodemographic characteristics – Household amenities – Morbidity and risk factors – Health care use – Mortality (all ages)	Ever-married women 15–49 years of age and never-married women15–49 years of age	– Birth history – Maternal and child health – Child mortality – Family planning and fertility preferences – Women’s and husbands’ background characteristics – Women’s employment status – Women’s autonomy and domestic violence – Marital and sexual relationships and living arrangements – Quality of health services – STIs and HIV/AIDS – NCDs and behavioural risk factors – Use of ICDS services	Men 15–54 years of age	– Reproductive history – Marital and sexual relationship, and living arrangements – Family planning and fertility preferences – Male involvement in health care – Quality of health services – NCDs and behavioural risk factors – Attitudes towards women’s autonomy and domestic violence – STIs and HIV/AIDS
**DLHS**						
DLHS-1	Household head	– Sociodemographic characteristics – Household amenities – Morbidity – Health care use – Child mortality – Maternal mortality	Currently-married women 15–44 years of age	– Birth history – Maternal and child health – Family planning – Quality of health services – STIs and HIV/AIDS	Men 20–54 years of age	– STIs and HIV/AIDS – Family planning
DLHS-2	Household head	– Sociodemographic characteristics – Household amenities – Morbidity – Mortality (all ages)	Currently-married women 15–44 years of age	– Birth history – Maternal and child health – Child mortality – Family planning – Quality of health services – STIs and HIV/AIDS	Husbands of eligible women	– STIs and HIV/AIDS – Family planning and fertility preferences
DLHS-3	Household head	– Sociodemographic characteristics – Household amenities – Health care use – Government health programmes – Mortality (all ages)	Ever-married women 15–49 years of age	– Birth history – Maternal and child health – Child mortality – Family planning and fertility preferences – Reproductive health – STIs and HIV/AIDS – Use of government health programmes	Not included in survey	N/A
			Never-married women 15–24 years of age	– Sex education and age at marriage – Reproductive health – STIs and HIV/AIDS		
DLHS-4	Household head	– Sociodemographic characteristics – Household amenities – Morbidity and behavioural risk factors – Health care use – Mortality (all ages)	Ever-married women15–49 years of age	– Birth history – Maternal and child health – Family planning and fertility preferences – Woman’s background characteristics – STIs and HIV/AIDS – Reproductive health – NCDs and behavioural risk factors	Not included in survey	N/A
**AHS**						
AHS baseline	Household head	– Sociodemographic characteristics – Household amenities - Morbidity and behavioural risk factors – Health care use – Mortality (all ages)	Ever-married women15–49 years of age	– Birth history – Maternal and child health – Birth registration	Not included in survey	N/A
			Currently married women 15–49 years of age	– Family planning and fertility preferences – STIs and HIV/AIDS – Awareness of childhood illness		
AHS 1st update	Household head	– Sociodemographic characteristics –Household amenities – Morbidity – Health care use – Mortality (all ages)	Ever-married women15–49 years of age	– Birth history – Maternal and child health – Birth registration	Not included in survey	N/A
			Currently married women 15–49 years of age	– Family planning and fertility preferences – STIs and HIV/AIDS – Awareness of childhood illness and danger signs in newborns		
AHS 2nd update	Household head	– Sociodemographic characteristics – Household amenities – Morbidity – Health care use – Mortality (all ages)	Ever-married women15–49 years of age	– Birth history – Maternal and child health – Birth registration	Not included in survey	N/A
			Currently married women 15–49 years of age	– Family planning and fertility preferences – STIs and HIV/AIDS – Awareness of childhood illness and danger signs in newborns		

AHS: Annual Health Survey; AIDS: acquired immunodeficiency syndrome; DLHS: District Level Household Survey; HIV: human immunodeficiency virus; ICDS: integrated child development services; N/A: not applicable; NCD: noncommunicable disease; NFHS: National Family Health Survey; STI: sexually transmitted infection.

The ever and/or currently married women interviewed in all surveys were of reproductive age; however, the age boundaries for inclusion varied both across surveys and between different rounds of the same survey. Women up to 49 years of age were selected as respondents in all rounds of the NFHS; the lower age limit for NFHS-1 was 13 years, which was raised to 15 years during subsequent rounds. Women aged 15–44 years were surveyed during the first two rounds of the DLHS and the upper age limit was raised to 49 years for ever-married women in DLHS-3 and DLHS-4. The age group for never-married women was 15–24 years in DLHS-3. The AHS surveyed women 15–49 years of age.

Male representation across surveys has been inconsistent. Only in four rounds have men been represented. Men aged 15–54 years were interviewed in NFHS-3 and NFHS-4. Men aged 20–54 years were interviewed in DLHS-1, whereas the husbands of eligible women, regardless of age, were interviewed in DLHS-2. The AHS did not interview men.

### Key survey themes

#### Interview

The key survey themes are shown in Table 2 and Table 3. The numbers of disease- or condition-specific questions increased in all surveys over time. From 246 to 868 questions in the NFHS, from 200 to 339 questions in the DLHS and from 137 to 207 questions in the AHS. Of these questions, more than 90.5% of questions were about maternal and child health and reproductive health (range: 90.6–99.1%; Table 3). Adult infections other than those of the reproductive tract received very little attention in the surveys, constituting only 0.6–3.0% of the total disease- or condition-specific questions. The number of questions on noncommunicable diseases increased for each round in the NFHS and DLHS, from two to 41 and zero to 10, respectively.

**Table 3:** Disease burden categories in major health surveys in India, 1992 to 2016

Survey	No. of questions^a,b^	No. (%)					
		Maternal and child health	Reproductive health issues other than infection^c^	Reproductive tract infection^d^	Other adult infections	NCDs	Injury
**NFHS**							
NFHS-1	246	123 (50.0)	118 (48.0)	0 (0.0)	3 (1.2)	2 (0.8)	0 (0.0)
NFHS-2	294	157 (53.4)	110 (37.4)	15 (5.1)	4 (1.4)	5 (1.7)	3 (1.0)
NFHS-3	694	254 (36.6)	313 (45.1)	71 (10.2)	10 (1.4)	22 (3.2)	24 (3.5)
NFHS-4	868	278 (32.0)	307 (35.4)	204 (23.5)	12 (1.4)	41 (4.7)	26 (3.0)
**DLHS**							
DLHS-1	200	105 (52.5)	57 (28.5)	32 (16.0)	6 (3.0)	0 (0.0)	0 (0.0)
DLHS-2	315	167 (53.0)	84 (26.7)	61 (19.4)	2 (0.6)	1 (0.3)	0 (0.0)
DLHS-3	385	165 (42.9)	153 (39.7)	57 (14.8)	9 (2.3)	1 (0.3)	0 (0.0)
DLHS-4	339	186 (54.9)	103 (30.4)	37 (10.9)	2 (0.6)	10 (2.9)	1 (0.3)
**AHS**							
AHS baseline	137	70 (51.1)	52 (38.0)	2 (1.5)	2 (1.5)	10 (7.3)	1 (0.7)
AHS 1st update	207	131 (63.3)	63 (30.4)	3 (1.4)	2 (1.0)	7 (3.4)	1 (0.5)
AHS 2nd update	207	131 (63.3)	63 (30.4)	3 (1.4)	2 (1.0)	7 (3.4)	1 (0.5)

AIDS: acquired immunodeficiency syndrome; AHS: Annual Health Survey; DLHS: District Level Household Survey; HIV: human immunodeficiency virus; NCD: noncommunicable disease; NFHS: National Family Health Survey.

^a^ Includes only questions on disease – or condition-specific – and excludes questions on background and sociodemographic characteristics, general health and health care.

^b^ Based on household, separate questionnaires for women and men.

^c^ Includes questions on family life education, family planning, fertility and reproductive preferences, and gender status and relations.

^d^ Includes questions on non-sexually and sexually transmitted infections including HIV/AIDS.

NFHS-4, DLHS-4 and the AHS baseline had questions on tobacco and alcohol use, which are major risk factors for chronic diseases. However, these questions did not fully meet the criteria for the STEPwise approach to surveillance, recommended by the World Health Organization (WHO) for monitoring risk factors over time.^22^^,^^23^ Only NFHS-3 and NFHS-4 had more than three questions related to injury (24 and 26 questions, respectively). However, all of these concerned intimate partner violence only (Table 3).

Questions on antenatal care, delivery and postnatal care, birth history and family planning were included in all surveys with the exception of postnatal care in NFHS-1. Key subthemes regarding child health were immunization, breastfeeding practices and common childhood morbidity symptoms (cough, fever and diarrhoea).

All rounds of the NFHS included questions on women’s employment status and fertility preferences. Rounds 2, 3 and 4 of the NFHS included questions on quality of health services, sexually transmitted infections (STIs), human immunodeficiency virus/acquired immunodeficiency syndrome (HIV/AIDS) and women’s autonomy. Several common themes were identified in the separate questionnaires completed by both women and men during NFHS-3 and NFHS-4: reproductive history; marital and sexual relationships; co-habitation; family planning and fertility preferences; quality of health services; STIs and HIV/AIDS. Additional themes in the men’s questionnaire were male involvement in health care and male attitudes towards women’s autonomy and domestic violence (Table 2).

DLHS-1 and DLHS-2 included questions on the quality of public sector health services; however, these were dropped in subsequent rounds. Several new themes were added to DLHS-3, including sex education, age at marriage, infertility, obstetric fistula, knowledge about reproduction and public sector health programmes; these were all dropped in DLHS-4. Additional information on fertility preferences and menstruation was documented in DLHS-3 and DLHS-4. The husbands’ questionnaire in DLHS-2 collected data on family planning and fertility preferences and on STIs and HIV/AIDS. In addition to the core themes of maternal and child health, birth registration was documented in the AHS.

#### Anthropometry and biomarkers

Height and weight were measured for children during all rounds of the NFHS, though the age varied in the different rounds (Table 4). Height and weight were measured for men and women in NFHS-2, NFHS-3 and NFHS-4. DLHS-1 and DLHS-3 did not include any anthropometric measurements. DLHS-2 included weight measurement only for children younger than 6 years to calculate weight-for-age as an indicator of nutritional status. Height and weight were measured in DLHS-4 and in a subsample of households in the AHS for children 1 month of age and older, as well as women and men.

**Table 4:** Anthropometry and biomarker measurements in three major health surveys in India, 1992 to 2016

Survey	Height and weight	Blood pressure	Blood test for:		
			Anaemia	HIV	Fasting plasma glucose
**NFHS**					
NFHS-1	– Children younger than 4 years	Not done	Not done	Not done	Not done
NFHS-2^a^	– Children younger than 3 years – Ever-married women 15–49 years of age	Not done	– Children 6–35 months of age – Ever-married women 15–49 years of age	Not done	Not done
NFHS-3	– Children younger than 5 years – Women 15–49 years of age – Men 15–54 years of age	Not done	– Children 6–59 months of age – Women 15–49 years of age – Men 15–54 years of age	– Women 15–49 years of age in a subsample of households – Men15–54 years of age in a subsample of households	Not done
NFHS-4^a^	– Children younger than 6 years – Women 15–49 years of age – Men 15–54 years of age in the subsample of households	– Women15–49 years of age – Men15–54 years of age in a subsample of households	– Children 6–71 months of age – Women 15–49 years of age – Men 15–54 years of age in a subsample of households	– Subsample of women 15–49 years of age in a subsample of households – Men 15–54 years of age in a subsample of households	– Women15–49 years of age – Men15–54 years of age in a subsample of households
**DLHS**					
DLHS-1	Not done	Not done	Not done	Not done	Not done
DLHS-2^b^	– Children younger than 6 years (weight only)	Not done	– Children younger than 6 years of age – Girls 10–19 years of age – Currently married pregnant women 15–44 years of age	Not done	Not done
DLHS-3	Not done	Not done	Not done	Not done	Not done
DLHS-4^a^	- Women, men and children 1 month or older	- Women and men 18 years or older	- Women, men and children 6 years or older	Not done	- Women and men 18 years or more of age
**AHS**					
All AHS^a^	- Women, men and children 1 month or older in a subsample of households	- Women and men 18 years or more of age in a subsample of households	- Women, men and children 6 months or older in a subsample of households	Not done	- Women and men 18 years or more of age in a subsample of households

AHS: Annual Health Survey; DLHS: District Level Household Survey; HIV: human immunodeficiency virus; NFHS: National Family Health Survey.

^a^ Testing of salt for iodine content was done for all households.

^b^ Testing of salt for iodine content was done in households that had maternal death.

The surveys evaluated various biomarkers, especially in the later rounds (Table 4). NFHS-2 included assessment of anaemia among children 6–35 months of age and ever-married women 15–49 years of age. Anaemia testing was also done for men in NFHS-3 and NFHS-4. Anaemia testing was done for children, girls and women in DLHS-2, but not in DLHS-3. DLHS-4 and the AHS included anaemia testing for children 6 months or older as well as women and men. HIV testing was included in a subsample of men and women in NFHS-3 and NFHS-4. Blood pressure measurement and blood testing for fasting plasma glucose were done in men and women in NFHS-4 and DLHS-4 and in a subsample of men and women in the AHS.

## Trend analyses

### Estimating child mortality

The information collected on deaths and age at death among all children born to ever-married women 15–49 years of age in their lifetime is consistent across all rounds of the NFHS, clarifying trends in child mortality over time using the lifetime data on births. In contrast, the data on birth histories varied in the different DLHS rounds, ranging from the preceding 3 years of the survey to lifetime data. The AHS baseline round collected birth history information for the preceding 3-year period, and the update rounds captured this information for the preceding year. On assessing the comparability of childhood mortality indicators across all rounds of the NFHS, DLHS and AHS, analogous estimates can be generated only for 3 years preceding the surveys for currently married women aged 15–44 years (Table 5).

**Table 5:** Birth history data for child mortality, maternal mortality and immunization across the three major health surveys in India, 1992 to 2016

Survey	Birth history for child mortality^a^	Maternal mortality	Immunization
**NFHS**			
NFHS-1	All births	Women13–49 years of age in the preceding 2 years	Last three live births in the preceding 4 years
NFHS-2	All births	Women 15–49 years of age in the preceding 2 years	Last two births in the preceding 3 years
NFHS-3	All births	Not available	All births in the preceding 5 years
NFHS-4	All births	Women 12 years of age in the preceding 2 years	All births in the preceding 5 years
**DLHS**			
DLHS-1	All births in the preceding 3 years	All women in the preceding 3 years	Last two surviving children born in the preceding 3 years
DLHS-2	All births	Women 15–44 years of age in the preceding 1 year	Last two surviving children born in the preceding 3 years
DLHS-3	All pregnancies in the preceding 3 years	Women 15–49 years of age in the preceding 3 years	Last two surviving children born in the preceding 3 years
DLHS-4	All pregnancies in the preceding 5–6 years	Women 15–49 years of age in the preceding 4 years	Last two surviving children born in the preceding 5–6 years
**AHS**			
AHS baseline	All pregnancies in the preceding 3 years	Women 15–49 years of age in the preceding 3 years	Last two surviving children born in the preceding 3 years
AHS update rounds	All pregnancies in the preceding year	Women 15–49 years of age in the preceding 1 year	Last two surviving children born in the preceding year

AHS: Annual Health Survey; DLHS: District Level Household Survey; NFHS: National Family Health Survey.

^a^ Birth includes only live births; pregnancy includes spontaneous abortions, induced abortions, live births and still births.

### Estimating maternal mortality

Comparable estimates of maternal deaths in the 2 years preceding the survey among women aged 15–49 years are possible using NFHS-1, NFHS-2 and NFHS-4, but maternal death data were not collected in NFHS-3. In the various rounds of the DLHS, the reference period for the collection of data on maternal deaths varies from 1 to 3 years preceding the survey. In the AHS, the reference period for maternal deaths ranges from 1 to 5 years preceding the last survey (Table 5).

### Immunization

Assessment of immunization trends over time using all the NFHS, DLHS and AHS rounds is possible only for the last two surviving children born in the 3 years preceding the surveys, due to variation in the reference periods and in the number of births and living children for which immunization data were collected in the various rounds (Table 5).

## Timeliness of data availability

Individual-level NFHS and DLHS data – without individual identifiers to maintain participants’ confidentiality – have to be made available in the public domain for analytical use. Table 6 (available at: http://www.who.int/bulletin/volumes/94/4/15-158493) shows the time between completion of data collection and release of individual-level data in the public domain. The time for the NFHS and DLHS to release their data varied between nine and 22 months. Until recently, only summary data had been reported for the AHS rounds. The individual-level data for the three AHS rounds were made available in November 2015, following 29 months of data collection for the second update round.

**Table 6:** Time lag for public availability of individual-level data from three major health surveys in India, 1992 to 2016

Survey	Data collection phase	Publicly available individual-level data	No. of months between data collection completion and publicly available individual-level data
**NFHS**			
NFHS-1	April 1992 to September 1993	August 1995	22
NFHS-2	November 1998 to December 1999	October 2000	9
NFHS-3	November 2005 to August 2006	September 2007	12
NFHS-4	March 2015 onwards	Data being collected as of February 2016	N/A
**DLHS**			
DLHS-1	May 1998 to October 1999	August 2001	21
DLHS-2	March 2002 to June 2005	August 2006	13
DLHS-3	December 2007 to December 2008	April 2010	15
DLHS-4	August 2012 to February 2014	December 2015	21
**AHS**			
AHS baseline	July 2010 to March 2011	November 2015	55
AHS 1st update	October 2011 to April 2012	November 2015	42
AHS 2nd update	November 2012 to May 2013	November 2015	29

AHS: Annual Health Survey; DLHS: District Level Household Survey; N/A: not applicable; NFHS: National Family Health Survey.

### Survey data publications

We identified 600, 95 and 73 publications resulting from the NFHS, DLHS and AHS respectively. Based on the review of the title and abstract, 337, 48 and three publications had used the NFHS, DLHS and AHS data, respectively; we reviewed the full text of these publications. Data from only NFHS-1 were used in 56 articles, data from NFHS-2 in 83 articles and data from NFHS-3 in 145 articles. The remaining 53 publications used data from two or more of the NFHS rounds. Only data from DLHS-2 and/or DLHS-3 were used in publications. No publication using DLHS-1 data was identified. One publication used AHS baseline survey data and two used the first update of the AHS survey data.

## Discussion

The national population-based health surveys in India started a quarter of a century ago with a predominant focus on maternal and child health, as these were considered the most visible and prominent health problems at that time. Over this period, the disease burden has shifted significantly towards noncommunicable diseases. Data from the global burden of disease study suggest that in India in 1990, diseases among children younger than 15 years and maternal disorders accounted for 57% of the total disease burden (with about 60% of this in the first year of life). In 2013, this burden had decreased to 33% of total disease burden, while noncommunicable diseases made up 52% of the total disease burden.^24^ However, in the latest national health surveys, questions on noncommunicable diseases constituted less than 5% of the total questions. Similarly, injuries are barely represented in national health surveys even though these contributed 13% of the total disease burden in 2013.^24^

While estimation of disease burden should not be the only criterion for inclusion in large-scale national health surveys, not having nationwide estimates for the conditions causing major disease burden is problematic. Reliable nationwide population-based data on major noncommunicable diseases, such as ischaemic heart disease, chronic obstructive pulmonary disease, stroke, low-back and neck pain and depression are scanty in India, as are similar data on injuries. Such data are also unavailable for tuberculosis and pneumonia.^25^

Attempts to improve coverage of noncommunicable diseases in national health surveys are a move in the right direction, but more could be done. The surveys could be expanded to meet WHO’s criteria for monitoring of noncommunicable diseases, the STEPwise approach to surveillance. This approach includes standardized data on four behavioural risk factors (tobacco use, alcohol use, low fruit and vegetable intake and physical inactivity) and four biological risk factors (body mass index, blood pressure, fasting blood glucose and blood cholesterol).^22^ Among the behavioural risk factors, tobacco and alcohol use are being assessed in national health surveys, but these do not fully meet the STEPS standardized data criteria. Low fruit and vegetable intake and physical inactivity are not yet being measured. Among the biological risk factors besides body mass index (which has been included in most surveys), blood pressure and fasting blood glucose have been added in the most recent rounds of the national surveys, but blood cholesterol is still not included. Recent national health surveys have only partly addressed these data gaps since our previous report, which preceded these surveys.^23^

National health surveys have the potential to increase data on disease burden by including biomarker measurements and diagnostic tests. For example, inclusion of HIV testing in NFHS-3 enabled a more accurate estimation of HIV prevalence.^26^^,^^27^ Rapid diagnostic tests for tuberculosis^25^^,^^28^ and malaria^29^ and assays for measuring blood lipids in the field^30^ could also be included. While a detailed assessment of all major diseases is not feasible in a single national survey, opportunities exist for adding additional categories of information. Some countries use a range of clinical and biomarker tests in their surveys and some regularly rotate the health and/or disease topics between rounds to make each round more manageable and frequent.^31^^–^^39^

Reliable cause-of-death data are important for informing decision-makers. India lacks an effective vital registration system that can provide such data across the country. To increase data on cause of death, automated algorithms could be used, which enable researchers to assign cause of death from large-scale verbal autopsy data. This is both more reliable and efficient than the resource-intensive physician-coding methods.^40^^,^^41^

Comparability of measurements over time and across population groups is fundamental to optimal interpretation and use of survey data.^2^^,^^42^^,^^43^ Given the enormous amount of data collected in national surveys, we calculated the feasibility of trends assessment over time for child mortality, maternal mortality and immunization between and within these surveys. All rounds of the NFHS had documented birth history consistently, allowing for comparable estimates over time as all births were captured with no restriction on reference period. However, the DLHS and AHS rounds captured births and/or pregnancies only for specific reference periods, which varied within and between surveys, thereby limiting the potential for using all the collected data for this purpose. Similarly, the reference period for data on immunization coverage varied within and between the surveys.

A systematic review reports that among publications in PubMed concerning global demographic and health surveys, there were many using the Indian NFHS data.^44^ We report 336 original research publications using NFHS data. On the other hand, the three DLHS rounds completed to date resulted in only 48 publications. This is puzzling, given that DLHS surveys were designed to provide district-level estimates, whereas the first three NFHS rounds, with smaller sample sizes, were designed only to provide state-level estimates. One of the reasons for the poorer use of DLHS could be that the data are made available in a format which is not user friendly. The AHS has provided individual-level data in the public domain only very recently, so the low number of publications from these data is not surprising.

The time between completion of data collection and individual-level data availability for analysis by researchers decreased between the first and third rounds of the NFHS and DLHS, but increased again for the last round of the DLHS. Part of the reason for this delay could be the effort needed to synchronize the DLHS-4 data with the AHS data, as these two surveys are complementary, with each covering approximately half the country’s population. In any case, such delays in use of a public good resource should be avoided. The recent availability of individual-level AHS data on request is a positive step towards increasing the effectiveness of the data.

## Conclusion

As national health surveys are resource-intensive, it would be wise to maximize the knowledge gained from them that could be used to improve population health in India. We propose that consultation – similar to the consultative development process underpinning the National Health Survey in Brazil^45^ – could improve the design of national health surveys in India. We have several recommendations. First, instead of having multiple, frequent surveys with overlapping goals, India should have a single major national health survey at five-year intervals. This could provide data on additional major causes of disease burden and their risk factors, along with cause-of-death data using automated verbal autopsy methods and include adult mortality rate estimation. The sample sizes should aim to provide state-level estimates for all indicators and district-level estimates for crucial indicators to capture the key features of health status heterogeneity across the country. Second, data collection on the key variables should be standardized to meet monitoring standards and to provide comparable data over time. Third, effective partnerships with a larger range of relevant stakeholders, including the academic community, should be established to increase the relevance and usefulness of the data. Fourth, detailed methods should be published. Fifth, individual-level data from these surveys should be made publicly available as soon as possible so that it can be used in the urgent tasks of informing policy and developing a more effective health system. Sixth, linking household survey data with health service use and administrative data, preferably using geospatial coding methods could be considered. Over time, India could also consider a continuous design for its national health survey, with advantages for survey management and timely provision of findings.^46^
